# Kinematic and EMG Responses to Pelvis and Leg Assistance Force during Treadmill Walking in Children with Cerebral Palsy

**DOI:** 10.1155/2016/5020348

**Published:** 2016-08-29

**Authors:** Ming Wu, Janis Kim, Pooja Arora, Deborah J. Gaebler-Spira, Yunhui Zhang

**Affiliations:** ^1^Sensor Motor Performance Program, Rehabilitation Institute of Chicago, Chicago, IL 60611, USA; ^2^Department of Physical Medicine and Rehabilitation, Northwestern University Medical School, Chicago, IL 60611, USA

## Abstract

Treadmill training has been used for improving locomotor function in children with cerebral palsy (CP), but the functional gains are relatively small, suggesting a need to improve current paradigms. The understanding of the kinematic and EMG responses to forces applied to the body of subjects during treadmill walking is crucial for improving current paradigms. The objective of this study was to determine the kinematics and EMG responses to the pelvis and/or leg assistance force. Ten children with spastic CP were recruited to participate in this study. A controlled assistance force was applied to the pelvis and/or legs during stance and swing phase of gait through a custom designed robotic system during walking. Muscle activities and spatial-temporal gait parameters were measured at different loading conditions during walking. In addition, the spatial-temporal gait parameters during overground walking before and after treadmill training were also collected. Applying pelvis assistance improved step height and applying leg assistance improved step length during walking, but applying leg assistance also reduced muscle activation of ankle flexor during the swing phase of gait. In addition, step length and self-selected walking speed significantly improved after one session of treadmill training with combined pelvis and leg assistance.

## 1. Introduction

Cerebral palsy (CP) is the most prevalent physical disability originating in childhood with an incidence of 2-3 per 1,000 live births [1]. Up to 90% of children with CP have difficulty in walking [2, 3]. Reduced walking speed and endurance are two of the main functional problems, particularly in children with more severe disabilities [4]. Locomotion plays a central role in healthy bone development [5] and children who are able to ambulate are more accomplished in activities of daily living and social roles, such as participation in the community, than children who use a wheelchair [6]. Thus, improving walking function is one key focus of clinical therapeutic interventions for children with CP.

Treadmill training has been used as a promising technique for improving locomotor function in children with cerebral palsy (CP) [7, 8]. However, while statistically significant improvements in walking function after treadmill training have been shown, the functional gains are relatively small [9, 10]. In addition, treadmill training requires a high level of involvement from a physical therapist [11]. In order to reduce therapist labor levels, several robotic gait training systems have been developed to provide robotic gait training to children with CP [12, 13]. These robotic systems are effective in reducing therapist labor during locomotor training but show relatively limited functional gains for some children with CP. For instance, a recent randomized study indicated that only a modest improvement in gait speed was obtained following a prolonged (20 sessions) robotic treadmill training using the pediatric Lokomat [14], suggesting a need for improving the efficacy of current treadmill training paradigm. Possible reasons why treadmill training may not be optimally effective for improving gait speed in children with CP include limitations of the current robotic systems, such as the lack of mediolateral movement of the pelvis, which may constrain the mediolateral movement of the pelvis during treadmill walking [15].

Weight shifting in the mediolateral direction is of one of key components of human locomotion [16]. However, this weight shifting ability is often impaired in children with CP compared to children who go through the normal stages of development [17]. For instance, children with CP were less efficient at weight shifting (demonstrated by a shorter range of motion of the center of pressure and slower velocity of the center of pressure displacement during visually guided weight shifting) than children with normal development. This impairment in weight shifting in children with CP may be related to weakness of hip abductors/adductors, which are suggested to play a crucial role in maintaining lateral balance during locomotion [18]. While the importance of weight shifting during locomotion of children with CP has been acknowledged, it remains unclear whether applying an assistance force to the pelvis during stance phase of gait will facilitate weight shifting and improve stepping.

Short step length is one of the key factors contributing to reduced walking speed of children with CP. Thus, in a clinical setting, assistance force is provided to the legs by physical therapists or robotic arms to facilitate leg swing during treadmill training in children with CP [9, 12]. While applying leg assistance may help to increase the step length of children with CP during treadmill training, applying too much assistance to the legs may be suboptimal to locomotor training because it may encourage passive instead of active participation of the subject; that is, the central neural system may reduce the motor output of subject in response to the assistance applied for the sake of the optimization of energy cost [19]. However, there is no evidence whether the muscle activation of the hip and/or ankle flexors of children with CP will be reduced when an assistance force is applied to legs during swing phase of gait.

In this study, we tested the spatial-temporal gait parameters and EMG responses to pelvis assistance applied during stance phase and leg assistance forces applied during swing phase in children with CP. We hypothesized that applying a lateral assistance load to the pelvis during the stance phase of gait will facilitate weight shifting, which will trigger an enhanced muscle activation of the hip abductors to stabilize the pelvis during the stance phase of gait, and applying a leg swing assistance force may reduce muscle activation of the hip flexors/ankle flexors during the swing phase of gait because the central nervous system may optimize energy output. In addition, we hypothesized that applying both leg assistance and pelvis assistance might improve step length and/or step height during treadmill walking. Furthermore, we tested the transfer of motor adaptation from the treadmill to overground walking after one session of robotic treadmill training. We hypothesized that the motor adaptation would be transferred from the treadmill to overground walking.

## 2. Methods

### 2.1. Subjects

Ten children (3 girls) with spastic CP were recruited to participate in this study. Mean age was 11 ± 3 years old. According to the Gross Motor Function Classification System (GMFCS) [20], 2 of them were classified as level I, 4 of them were classified as level II, and 4 of them were classified as level III; see Table 1 for details.

Inclusion criteria were as follows: (a) age 7–16 years old; (b) spastic CP; (c) without Botulinum toxin treatment or surgery within 3 months before the onset of the study; (d) GMFCS levels that were I to III; (e) ability to signal pain, fear, or discomfort reliably; (d) ability to ambulate for at least 10 meters with/without assistive device.

Exclusion criteria were as follows: (a) severe lower extremity contractures, fractures, osseous instabilities, and osteoporosis; (b) unhealed skin lesions in the lower extremities; (c) thromboembolic diseases, cardiovascular instability, and aggressive or self-harming behaviors.

### 2.2. Apparatus

**Figure 1:**
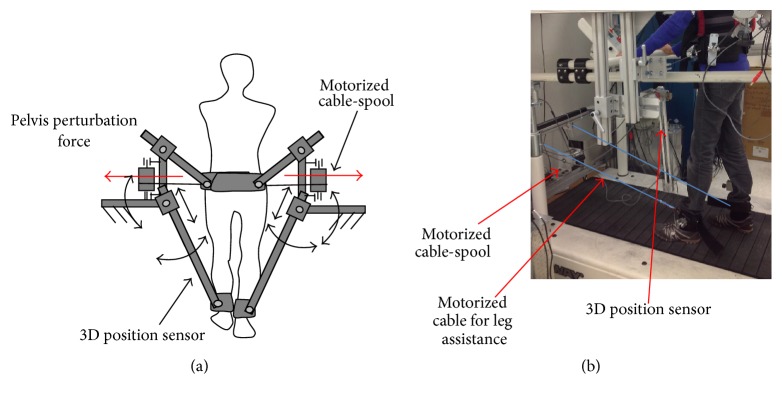
This figure illustrates the 3D cable-driven apparatus that was used with a treadmill and body weight support system. Four cables driven by four motors, pulleys, and cable spools were used to apply pelvis and leg assistance loads during treadmill walking. A personal computer was used to control the loads produced by four motors, applying controlled forces at targeted phase of gait.

A custom designed 3D cable-driven gait training system was used to apply controlled assistance forces to the pelvis and legs, to facilitate weight shifting and leg swing, respectively, during treadmill walking; see Figure 1. The cable-driven robotic gait training system for leg assistance has been reported previously [21]. In brief, four nylon-coated stainless-steel cables (diameter 1.6 mm), which are driven by four motors and cable spools (two of them are located at the frontal of the treadmill and other two motors are located at the back of the treadmill), are affixed to custom braces that are strapped to the subject's legs to provide a controlled assistance (pulling forward) forces to legs. In this study, additional two motors (AKM33H, Kollmorgen, Drive amplifier, Servostar 30661), which are attached to the frame located at the side of treadmill, were used to provide controlled assistance forces at the pelvis in the mediolateral direction. Additionally, two sets of custom designed 3D position sensors were attached to the pelvis and legs above ankle through a strap and were used to record the pelvis and leg positions. Specifically, each position sensor consists of a detector bar and three potentiometers. One linear potentiometer (SP-2, Celesco, Chatsworth, CA) was used to measure the linear movement of the bar and the other two rotational potentiometers (P2201, Novotechnik, Southborough, MA) were used to measure rotational movements of the bar in the anterior-posterior and medial-lateral directions [21]. The cable driven system is compliant and highly backdrivable [21], which allows subjects to freely move their pelvis and legs through a natural gait pattern.

### 2.3. Protocol

An overhead harness was used while subjects walked on a treadmill. Body weight support was provided for one subject to prevent knee buckling or toe dragging and no body weight support was provided for other 9 subjects. The treadmill speed was set at each subject's comfortable speed determined at the beginning of experiment. Subjects were allowed to hold onto the bar in front of them for the sake of safety and to wear their orthosis. Each subject participated in two test sessions. Specifically, in session 1, subjects walked on a treadmill without a load for one minute, that is, baseline. Then, subjects walked on a treadmill with 3 loading conditions, that is, (1) pelvis assistance load only, (2) leg assistance load only, and (3) combined pelvis and leg assistance load. The order of these three loading conditions was randomized across subjects. Subjects walked for 3 minutes in each loading condition with a one-minute standing break inserted in between two test conditions. The peak value of the pelvis assistance force was set at ~14% of body weight, and the peak leg assistance force was set at ~6% of body weight, although these peak forces were adjusted based on the tolerance of each subject (i.e., to make sure they felt some challenges but not too overwhelming when the force was applied); see Table 2. The ankle position signals were used to trigger pelvis and ankle loading at targeted phases of gait. Specifically, toe-off was defined as the time during which the ankle position, which was measured using ankle position sensor, changed its moving direction from backward to forward; heel-contact was defined as the time during which the ankle position changed its moving direction from forward to backward [22, 23]. The leg assistance load was applied bilaterally to the ankle starting from toe-off to mid-swing, and the pelvis assistance load was applied bilaterally in the lateral direction (for facilitating weight shifting) starting at heel contact to mid-stance of the ipsilateral leg, determined based on signals recorded by ankle position sensors.

After subjects completed all the test conditions of session 1, subjects were given a 5-minute sitting break. Then, session 2 was initiated, in which subjects walked on a treadmill while a controlled assistance load was applied to both the pelvis and leg for another 20 minutes using a protocol similar to condition 3 of session 1. Short standing breaks were allowed as necessary depending on the tolerance of each subject during the test. Self-selected and fast (i.e., subjects were instructed to walk as fast as they could without running) overground walking speeds were tested using the GaitRite (CIR Systems Inc., Sparta, NJ), a 4.3 m long mat with embedded pressure sensors for measuring spatiotemporal gait parameters, before and after treadmill training. Three trials were tested for each speed and were averaged across the 3 trials for each test condition. Spatiotemporal parameters, including step length, stance time, and swing time, during self-selected and fast overground walking were also calculated using the GaitRite software and averaged across the three trials and two legs. The kinematics of the pelvis and legs during treadmill walking were recorded using two sets of custom-designed positional sensors, mentioned previously, and sampled at 500 Hz using a data acquisition card (National Instruments, Austin, TX, USA) on a personal computer. Custom LabVIEW (National Instruments) software was used for controlling the data acquisition and sending motor commands to the motor drivers at targeted phase of gait, which was determined using a custom designed ankle positional sensor.

The EMG activity of the right Tibialis Anterior (TA), Medial Gastrocnemius (MG), Soleus (SO), Vastus Medialis (VM), Rectus Femoris (RF), Medial Hamstrings (MH), hip Adductors (ADD), and Abductors (ABD) was recorded for all the testing sessions during treadmill walking (for the convenience of setup, only the right leg's muscle activities were recorded, although pelvis and leg assistance forces were applied bilaterally). Active Delsys electrodes (Model De-2.1, Delsys Inc. Boston, MA, USA) were applied to lightly abraded, degreased skin over the respective muscle belly. The leads were attached to a preamplifier/filter system (amplification ×1,000) and all signals were band-passed filtered (20–450 Hz), and sampled at 500 Hz on a personal computer, which was synchronized with the computer used for recording kinematic data.

### 2.4. Data Analysis

All kinematic data were smoothed using a 4th order Butterworth low-pass filter (cut-off frequency: 8 Hz) with zero lag (The MathWorks, Natick, Massachusetts). Step length and step height during treadmill walking were derived from subject's ankle trajectory, which were recorded using two ankle position sensors [24]. Step length was quantified as the horizontal distance between the two legs' ankle positions at the timing of heel-contact. Step height was quantified as the vertical difference between the leg's highest ankle position and lowest ankle position during a gait cycle. Swing time was quantified as the time between toe-off and heel-contact normalized to the gait cycle time. Weight shifting in the frontal plane during treadmill walking was quantified using the minimal lateral distance between the center of the pelvis and ankle position of the supporting leg, which was calculated using signals recorded by a set of pelvis and leg positional sensors. The spatial temporal parameters of the last 10 steps during treadmill walking of each loading condition were averaged and further averaged across both legs.

The EMG signals were notch filtered at 55 Hz to 65 Hz using 4th order Butterworth filter and rectified. Then, these data were segmented into step cycle from heel contact to next heel contact, dependent on the measured ankle position using the position sensor. The last ten steps of each condition were used for analysis. Due to the variability in step duration from cycle to cycle, the data from each cycle were interpolated and resampled and then averaged across 10 steps to create a mean EMG pattern, which were smoothed using low-pass filter at 40 Hz using 4th order Butterworth filter. The EMG data of each muscle from each condition were normalized to the average peak value of that muscle's activation pattern for the condition with maximum treadmill walking speed of each participant. Smoothed EMGs were integrated for the targeted period of the gait cycle, that is, from late stance (~10% gait cycle before toe off) to mid-swing and from heel contact to mid-stance. Spatial-temporal gait parameters during overground walking were obtained using data collection software of GaitRite (CIR Systems Inc., Sparta, NJ).

Repeated measures ANOVAs were used for the effect of different loading conditions on the spatial-temporal gait parameters and integrated EMG areas during treadmill walking, with significance noted at *p* < 0.05. If the ANOVA revealed significant differences, Tukey-Kramer post hoc tests were used to identify specific differences between different conditions, again with significance noted at *p* < 0.05. In addition, the spatial-temporal parameters and gait speeds during overground walking were also compared before and after treadmill walking to identify the transfer effect, again, with significance noted at *p* < 0.05.

## 3. Results

**Figure 2:**
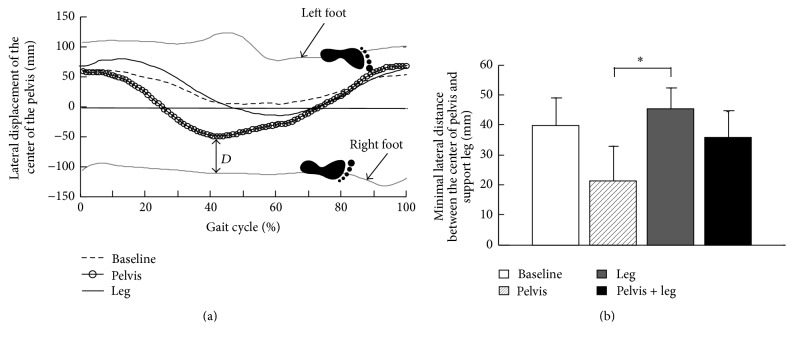
Displacement of the center of the pelvis in the mediolateral direction while subjects walking on a treadmill. (a) Displacement of the center of the pelvis from one child with CP with no load, that is, baseline, with pelvis assistance, and leg assistance. Displacements of the center of the pelvis shown in the figure were average of 10 strides for each condition. Gray curves located at the top and the bottom indicated the trajectories of the left and right feet during treadmill walking. All the displacements of the center of the pelvis were normalized to the gait cycle, starting from heel strike of the right leg. “*D*” indicates the minimal distance in the mediolateral direction between the center of the pelvis and supporting leg during stance. (b) Group average of the minimal distance between the center of the pelvis and supporting leg across 9 subjects at 4 different loading conditions. The bar and error bar indicate the mean and standard deviation of the minimal distance across 10 subjects for each loading condition. Asterisk (*∗*) indicates significant effect of loading conditions.

Applying controlled pelvis assistance facilitated weight shifting, indicated as a reduced minimal lateral distance between the pelvis and the supporting leg during stance phase. The displacements of the center of the pelvis in the mediolateral direction, from one child with CP, with no load, that is, baseline, and with pelvis or leg assistance load are shown in Figure 2(a). We observed a greater lateral shifting of the center of mass (estimated using the center of the pelvis) to a position closer to the lateral limit of the base of support of the right leg when a pelvis assistance load was applied but a modest change in lateral shifting of the center of the mass in comparison with the baseline when a leg assistance load was applied. Results from all subjects indicated that loading condition had a significant impact on the minimal lateral distance between the center of the pelvis and ankle position of the supporting leg during stance (*p* = 0.02, *n* = 9, ANOVA, data from one child was excluded because the pattern of data across different load conditions was distinct from all other subjects due to unclear reasons). Post hoc tests indicated significant differences in the minimal lateral distance between the conditions with pelvis assistance only versus leg assistance only (*p* = 0.01) but indicated no significant difference between the conditions with baseline versus leg assistance only (*p* = 0.85), the conditions with baseline versus pelvis assistance only (*p* = 0.08), and the conditions with baseline versus combined pelvis and leg assistance (*p* = 0.99), Figure 2(b).

**Figure 3:**
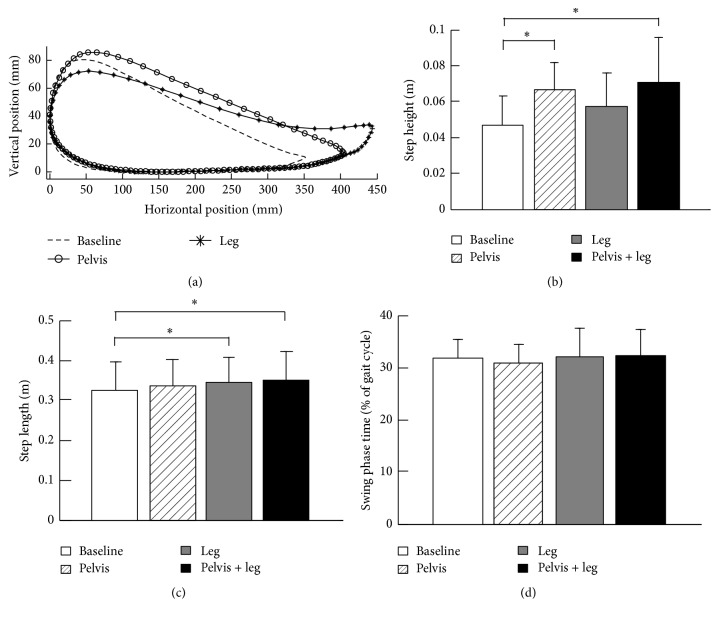
(a) Ankle trajectories in the sagittal plane are shown for one child with CP during treadmill walking with no load, that is, baseline, with pelvis and leg assistances. The ensemble-average trajectories across 10 strides are shown for each loading condition. Group averages of step height (b), step length (c), and swing time (d) during treadmill walking with different loading conditions. The bar and error bar indicate the mean and standard deviation of spatial and temporal gait parameters across 10 subjects for each loading condition. Asterisk (*∗*) indicates significant effect of loading conditions.

Applying a controlled pelvis and/or leg assistance force during treadmill walking had impact on the step length and height of children with CP. Ankle trajectories in the sagittal plane, from one child with CP, with no load, and with pelvis assistance or leg assistance load are shown in Figure 3(a). We observed an increase in step height when pelvis assistance was applied and an increase in step length when leg assistance was applied; see Figure 3(a). Results from a group of children with CP indicated that applying a controlled pelvis and/or leg assistance force had a significant impact on the step height (*p* = 0.01, *n* = 9, the step height from 1 subject was excluded because the data point was classified as an outlier, defined as a magnitude that was >3 SD above the population mean). Post hoc tests indicated significant differences in step height between baseline and the condition with pelvis assistance only (55.7% increase, *p* = 0.04) and between baseline and the condition with combined pelvis and leg assistance (68.0% increase, *p* = 0.01) but no significant difference between baseline and the condition with leg assistance only (31.6% increase, *p* = 0.44) (Figure 3(b)). In addition, group results indicated that pelvis and/or leg assistance loads had a significant impact on step length of children with CP (*p* = 0.01, *n* = 10, ANOVA). Post hoc tests indicated significant differences in step length between baseline and the condition with combined pelvis and leg assistance (9.2% increase, *p* = 0.03) and between baseline and the condition with leg assistance only (8.2% increase, *p* = 0.01) but no significant difference between baseline and the condition with pelvis assistance only (5.4% increase, *p* = 0.28); see Figure 3(c). Group results indicated that applying pelvis and/or leg assistance had no significant impact on the swing time (*p* = 0.70) (Figure 3(d)).

**Figure 4:**
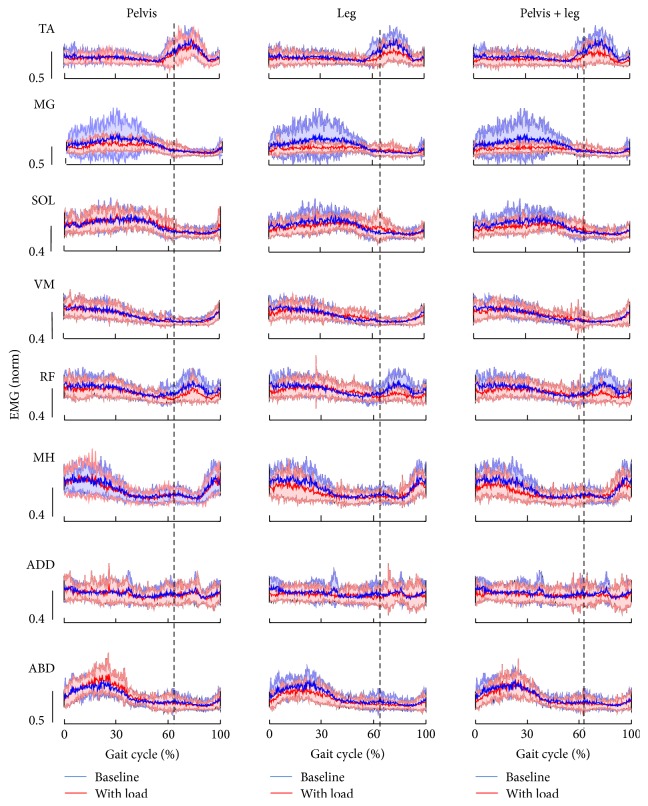
EMG activity patterns from 8 muscles of the right leg during treadmill walking with 3 different loading conditions, that is, pelvis assistance only, leg assistance only, and combined pelvis and leg assistance, in comparison with baseline condition in children with CP are shown. EMG patterns were averaged over the last 10 steps with load for each subject and were further averaged across 10 subjects for all 8 muscles except for MG (*n* = 9). EMG data of MG from one subject were excluded from average because the data was considered as an outlier. In all graphs, thick lines with surrounding thin lines represent the mean and standard deviation of EMG data. EMG data were normalized to the peak values of each muscle with subjects walking on a treadmill with maximum walking speed of each subject. All EMG signals were normalized in time to 100% of the gait cycle. Dashed vertical line indicates the onset of the swing phase of gait.

**Figure 5:**
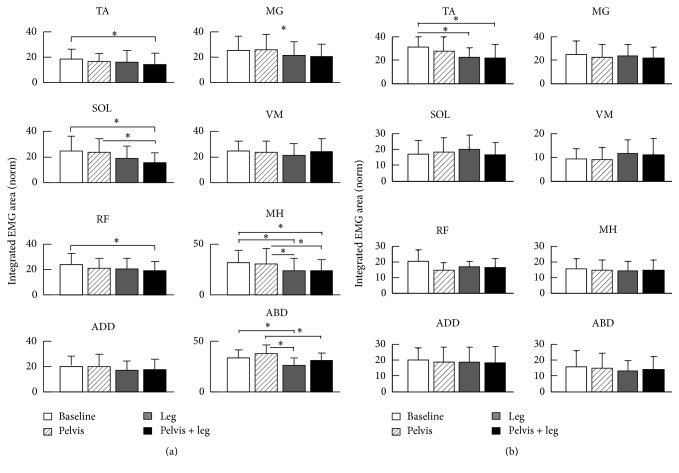
Average of integrated EMG area during stance phase (i.e., from heel strike to mid-stance, when the pelvis assistance force was applied), (a), and during swing phase (i.e., from late stance, 10% before toe-off, to mid-swing), (b), at 4 different loading conditions, that is, baseline, pelvis assistance only, leg assistance only, and combined pelvis and leg assistance, is shown. The bar and error bar indicate the mean and standard deviation of the integrated EMG area across subjects for each load condition. Asterisk (*∗*) indicates significant effect of treatment.

Applying a controlled pelvis and/or leg assistance force induced changes in the leg muscle activity pattern. Specifically, applying a leg assistance force induced a decrease in the magnitude of TA during swing phase of gait and a slight decrease in magnitude of MG during stance phase of gait; see Figure 4. In addition, applying a pelvis assistance force induced an increase in the magnitude of ABD during stance phase of gait but had a modest impact on the muscle activities of ADD; see Figure 4. A group average of integrated muscle activities during the early stance phase (from heel contact to mid-stance) and early swing phase (from late stance to mid-swing) are shown in Figure 5. The loading condition had a significant impact on the integrated muscle activity of TA during swing phase (*p* = 0.002). Post hoc tests indicated a significant decrease in muscle activity of TA from baseline to the conditions with leg assistance (*p* = 0.006) and with combined pelvis and leg assistance (*p* = 0.003). The loading condition had no significant impact on the integrated muscle activity of other muscles during swing phase (*p* = 0.55 for MG, *p* = 0.32 for SOL, *p* = 0.14 for VM, *p* = 0.08 for RF, *p* = 0.85 for MH, *p* = 0.76 for ADD, and *p* = 0.14 for ABD). In addition, loading condition had a significant impact on the integrated muscle activity of TA during stance phase of gait (*p* = 0.04). Post hoc tests indicated a significant decrease in muscle activity of TA from baseline to the condition with combined pelvis and leg assistance (*p* = 0.03). The loading condition had a significant impact on the integrated muscle activity of MG (*p* = 0.02) and ADD (*p* = 0.02) during the stance phase of gait, although post hoc tests indicated no significant differences between the other two conditions for these two muscles (*p* > 0.05). The loading condition had a significant impact on the integrated muscle activity of SOL during stance phase of gait (*p* = 0.01). Post hoc tests indicated a significant difference between baseline versus combined pelvis and leg assistance (*p* = 0.02) and pelvis only versus combined pelvis and leg assistance (*p* = 0.04). No significant differences were observed between the other two conditions (*p* > 0.05). Loading condition had no significant impact on the integrated muscle activity of VM during stance phase of gait (*p* = 0.48). The loading condition had a significant impact on the integrated muscle activity of RF during the stance phase of gait (*p* = 0.04). Post hoc tests indicated a significant decrease in muscle activity of RF from baseline to the condition with combined pelvis and leg assistance (*p* = 0.03). No significant differences were observed between other conditions (*p* > 0.05). The loading condition had a significant impact on the integrated muscle activity of MH during the stance phase of gait (*p* < 0.001). Post hoc tests indicated significant differences between baseline versus combined pelvis and leg assistance (*p* < 0.001), baseline versus leg assistance only (*p* < 0.001), pelvis versus leg assistance only (*p* = 0.002), and pelvis versus combined pelvis and leg assistance only (*p* = 0.002). No significant differences were observed between other conditions (*p* > 0.05). The loading condition had a significant impact on the integrated muscle activity of ABD during the stance phase of gait (*p* < 0.001). Post hoc tests indicated significant differences between baseline versus leg assistance only (*p* = 0.02), pelvis assistance only versus leg assistance only (*p* < 0.001), and pelvis assistance only versus combined pelvis and leg assistance (*p* = 0.02). No significant differences were observed between the other two conditions (*p* > 0.05).

**Figure 6:**
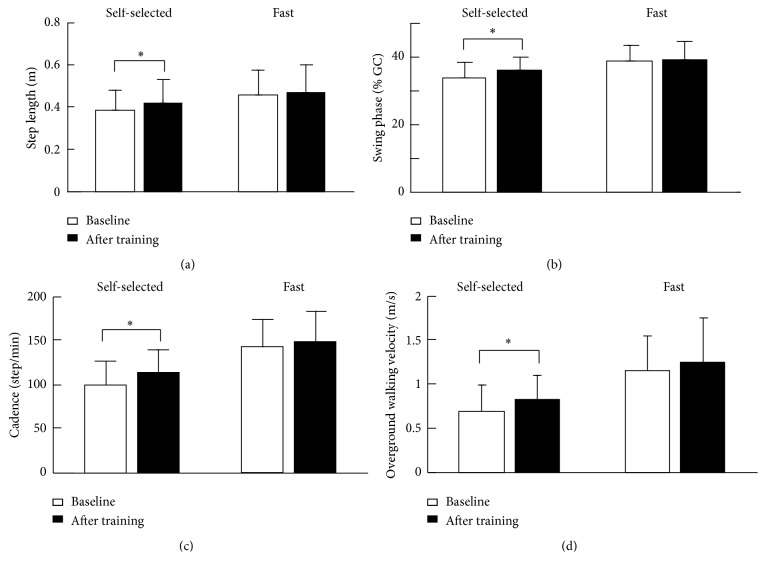
Step length, (a), self-selected and fast overground walking speeds, (b), step frequency (c), swing phase ratio, (d), and single leg support time, (d), in children with CP before and after treadmill training with controlled assistance forces applied to the pelvis and legs through the 3D cable-driven robot. The bar and error bar indicate the mean and standard deviation of the spatial and temporal gait parameters across 10 subjects. Asterisk (*∗*) indicates significant effect of treatment.

Step length during overground walking at a self-selected speed significantly increased (9.6% increase) after one session of treadmill training (~20 minutes) with combined pelvis and leg assistance force (*p* = 0.02), suggesting that a potential transfer of motor skills occurred from robotic-assisted treadmill training to overground walking in children with CP; see Figure 6(a). Swing phase time (normalized to gait cycle time, *p* = 0.01) and step cadence (*p* = 0.001) during overground walking at a self-selected speed also significantly increased after one session of treadmill training; see Figures 6(b) and 6(c). In addition, self-selected overground walking speed significantly increased (22.2%) after one session of treadmill training with combined pelvis and leg assistance (*p* = 0.001); see Figure 6(d).

Step length during overground walking with fast speed had no change (1.7% increase) after one session of treadmill training with a controlled pelvis and leg assistance force (*p* = 0.55); see Figure 6(a). In addition, there were no significant changes in swing phase time during overground walking at a fast speed after one session of treadmill training (*p* = 0.66); see Figure 6(b). Fast walking speed had no significant change (8.3% increase, *p* = 0.22) after treadmill training; see Figure 6(d).

## 4. Discussion

Applying a controlled pelvis assistance force improved step height, and applying a controlled leg assistance force improved step length in children with CP, although leg assistance reduced muscle activation of leg flexors and pelvis assistance tended to increase muscle activation of hip abductors. Furthermore, we observed a significant increase in step length and self-selected walking speed of children with CP during overground walking after one session of treadmill training in which controlled forces were applied to the pelvis and leg, suggesting a potential transfer of motor adaptation from the treadmill to overground walking.

Improved weight shifting facilitated by the pelvis assistance force during treadmill walking may improve stepping in children with CP. To the best of our knowledge, this is the first study that provided direct evidence that applying lateral pelvis assistance to facilitate weight shifting in children with CP may improve step height in children with CP. Many children with CP have impairments in weight shifting [25, 26], which has been shown to be correlated to their walking speed [25]. One possible reason for insufficient weight shifting in children with CP may be due to muscle weakness of the hip abductors and adductors [27, 28], which have been suggested to play crucial roles in maintaining lateral balance control during locomotion [18]. The hip abductors and adductors serve as a stabilizer for the ipsilateral lower extremity during single leg stance. Therefore, weakness of the hip abductors and adductors may limit a child with CP to bear weight on one leg for a prolonged period of time, which may result in a shortened swing time and step length on the contralateral leg. In addition, a child with CP may have a limited capacity to laterally shift their center of mass to a position closer to the lateral limit of the base of support in children with CP when weakness in the hip abductors and adductors is present. Insufficient weight shifting to the ipsilateral leg may induce an inefficient unloading of the contralateral leg, a key afferent component for modulating transition from stance to swing [29, 30]. In the current study, an assistance load applied to the pelvis during stance also triggered enhanced muscle activity of the hip abductors (16% increase) (Figure 4), although this was not significant due to variability of integrated muscle activity across subjects (*p* = 0.3); see Figure 5. Thus, it is feasible that the controlled assistance force applied to the pelvis in the mediolateral direction may facilitate weight shifting in children with CP during the stance phase of gait, indicated as a reduction in minimal distance in the mediolateral direction between the center of the pelvis and supporting leg during stance, although this was not significant due to large variability across subjects. One mechanism through which gait pattern improvements in children with CP may occur is that an improvement in weight shifting to the ipsilateral leg (i.e., standing leg) may facilitate unloading of the contralateral leg (i.e., swing leg). Unloading afferent input from the swing leg may facilitate leg swing [29, 30]. We observed significant improvements in step height and step length, in children with CP with the application of a pelvis assistance force.

The central nervous system of children with CP may adapt to an external assistance force applied to the leg(s) during the swing phase of gait. In the current study, the muscle activity of the TA of the ipsilateral leg (during swing phase) and MH and ABD of the contralateral leg (during stance phase) significantly decreased (the integrated muscle activity decreased 24.4%, for TA, 29.3%, for MH, and 18.4% for ABD, resp., in comparison to baseline) when a controlled assistance force was applied to both legs during the swing phase. This reduction in muscle activity suggests that the central neural system of children with CP recalibrated the motor output of the leg muscles in response to the externally applied leg assistance force, probably due to the optimization of the energy cost [19]. This is consistent with previous studies in which an ankle assistance force induced a reduction in muscle activity when an assistance force was applied to assist ankle dorsiflexion of healthy adults [31]. Given that the activity of the central neural system of children with CP may decrease when an external assistance force is applied, a large leg assistance force (e.g., with the magnitude of force at 6% of body weight) may be suboptimal for motor learning during locomotor training in children with CP because it may encourage passive rather than active training. Previous studies have indicated that active training is more effective than passive training [32, 33]. This is also consistent with a previous clinical study, which indicated that only a modest increase in the gait speed of children with CP was observed after robotic training in which a passive guidance force was applied to both legs [14].

The locomotor skills obtained during robotic treadmill training may partially transfer to overground walking in children with CP. While differences in the kinetics between treadmill and overground walking have been observed [34], the differences in the kinematics [35] between these two walking conditions are generally small in children with CP. The neural circuits controlling locomotion during treadmill and overground walking may be partially overlapped [36]. Thus, the motor skills obtained during treadmill training may partially transfer to overground walking in children with CP, which is consistent with previous studies in patients with spinal cord injury [24] and patients after stroke [36]. However, many other factors may influence the transfer of motor skills from training tasks to application tasks. For instance, visual information is quite different between treadmill and overground walking. During overground walking, subjects are moving through space and visual information is changing, whereas this is not happening during treadmill walking. In addition, subjects held on to a static bar during treadmill walking but used a walker/crutch or no assistive device during overground walking, which can affect the subject's stability and therefore walking pattern. In this study, the increase in step length during overground walking (9.6%) at a self-selected walking speed is comparable to the increase in step length during treadmill walking (9.2%) with combined pelvis and leg assistance versus baseline, suggesting that a majority of motor skills may be transferred from treadmill training to overground walking in children with CP.

In addition, while no significant change was seen in swing time with combined pelvis and legs assistance during treadmill walking, significant increase was observed during overground walking after robotic treadmill training. The increase in swing time, suggesting an improvement in balance of the support leg, after treadmill training may be due to the training effect of the mediolateral assistance force applied to the pelvis during treadmill training. The pelvis assistance force may facilitate weight shifting and children with CP may be forced to use more of the hip abductor/adductors, which provide crucial contributions to frontal plane balance control during walking [18], during robotic treadmill training. Thus, repetitive exposure to a pelvis assistance force during treadmill training may be helpful for improving motor control of the hip abductor/adductors. An increase in swing time may allow children with CP to take a longer step, paired with an increase in cadence, resulting an improvement in walking speed after robotic treadmill training.

This study may have some potential clinical applications. For instance, results from this study indicate that applying an assistance force to the pelvis during the stance phase of gait may facilitate weight shift and improve stepping in children with CP. Thus, it would be helpful for physical therapists to apply assistance forces to the pelvis of children with CP (even for these patients with GMFM level at I to III) during treadmill training. On the other hand, results from this study indicated that a leg assistance that is too large (e.g., at 6% of body weight) is suboptimal to encourage active participation of children with CP during treadmill training and may instead result in a decrease in leg muscle activity. Thus, results from this study suggest that physical therapists refrain from applying a large (e.g., 6% of body weight) leg assistance during treadmill training in order to encourage active participation of children with CP.

This study has several limitations. For instance, we did not test the retention of the improvements observed in step length and walking speed after 20 minutes of treadmill training with combined pelvis and leg assistance. We do not know how long these improvements will be retained, but we expect that long-term (e.g., 6 weeks) treadmill training using the 3D cable-driven robotic system may induce functional improvements in walking speed and endurance in children with CP. We do not know whether the magnitude, the timing, and the duration of force were optimal for each subject. Further studies are warranted to examine the effect of these parameters of the force perturbation on the kinematics of the pelvis and leg and EMG responses in children with CP. We did not collect the information about the level of spasticity of subjects who participated in this study. Thus, we do not know to what extent the level of spasticity had an impact on the gait performance of these subjects we tested [37]. We did not measure muscle activities during overground walking. In addition, the functional level of children with CP may also have an impact on improvements in walking speeds and step length after training. In this study, the subjects' GMFCS levels were I to III and all subjects could ambulate with/without assistive device. We do not know whether subjects with lower functional levels, such as GMFCS levels IV, and subjects who cannot ambulate will also have responses similar to those observed in this study. Subject's age may also impact the improvements in walking speeds and step length/step height after training. For instance, older healthy children may be faster to adapt gait than younger children [38]. However, we observed no correlation between the changes in step length and height and the age of subjects (*p* = 0.45 for step length, *p* = 0.74 for step height), although we observed a trend that older children with CP had more improvements in overground walking speed after robotic treadmill training than younger children with CP, but this was not significant; that is, *p* = 0.07. Thus, other factors, such as functional level of subjects, might also have impact on these functional gains.

## 5. Conclusion

Improved weight shifting induced by a robotically applied pelvis assistance force during stance may facilitate stepping in children with CP during treadmill walking. In addition, applying a large leg swing assistance force (e.g., with the magnitude more than 6% of body weight) during treadmill training may reduce the active participation of children with CP. In particular, motor skills obtained during treadmill training may partially transfer to overground walking in children with CP. Results from this study may be used to develop a robotic gait training paradigm for improving walking function in children with CP through long-term robotic gait training in clinical settings.

## Figures and Tables

**Table 1:** Subject information indicating age, type of CP, weight, gender, GMFCS level, and orthosis and/or assistive devices used by subjects at the time of the study. RRW: reverse rolling walker; AFO: ankle-foot orthosis.

Number	Age (year)	Body weight (kg)	Gender	GMFCS level	Type of CP	Orthosis and assistive devices
1	7	22.5	M	III	Spastic diplegia	Bilateral AFO/crutches
2	10	27.0	M	II	Spastic diplegia	Bilateral AFO/none
3	11	34.7	M	III	Spastic diplegia	Bilateral AFO/RRW
4	16	58.5	F	I	Spastic diplegia	None/none
5	12	47.3	M	II	Spastic diplegia	Bilateral AFO/none
6	14	42.3	M	II	Spastic diplegia	Bilateral AFO/none
7	10	25.2	M	I	Spastic diplegia	None/none
8	9	27.5	F	III	Spastic diplegia	Bilateral AFO/RRW
9	9	43.7	M	II	Spastic diplegia	Orthotics/none
10	12	54.4	F	III	Spastic diplegia	Bilateral AFO/RRW

**Table 2:** Testing parameters indicating body weight support, test speed, and average peak assistance forces applied to the leg at the ankle and pelvis. BW: body weight.

Number	Treadmill speed (m/s)	Pelvis assistance force (N)	Leg assistance force (N)	Body weight support (% BW)
1	0.37	52 (23.6%)	17 (7.7%)	0
2	0.48	41 (15.5%)	22 (8.3%)	0
3	0.18	52 (15.3%)	22 (6.4%)	30%
4	0.76	66 (11.5%)	22 (3.8%)	0
5	0.46	40 (8.6%)	23 (5.0%)	0
6	0.36	41 (9.9%)	23 (5.5%)	0
7	0.23	48 (19.4%)	20 (8.1%)	0
8	0.51	43 (15.9%)	17 (6.3%)	0
9	0.78	51 (11.9%)	27 (6.3%)	0
10	0.30	46 (8.6%)	28 (5.2%)	0

Mean	0.44 ± 0.20	48.0 ± 7.9 (14.0 ± 4.9%)	22.1 ± 3.6 (6.3 ± 1.4%)	

## Additional Points

*Highlights*. (1) Applying a pelvis assistance force may facilitate weight shifting and improve step height. (2) Applying leg assistance may improve step length but also may reduce muscle activation of the leg flexors. (3) Motor skills obtained during treadmill training may transfer to overground walking in children with CP.

## References

[1] Rosen M. G., Dickinson J. C. (1992). The incidence of cerebral palsy. *American Journal of Obstetrics & Gynecology*.

[2] Hutton J. L., Pharoah P. O. D., Rosenbloom L. (2002). Effects of cognitive, motor, and sensory disabilities on survival in cerebral palsy. *Archives of Disease in Childhood*.

[3] Pharoah P. O. D., Cooke T., Johnson M. A., King R., Mutch L. (1998). Epidemiology of cerebral palsy in England and Scotland, 1984–1989. *Archives of Disease in Childhood: Fetal and Neonatal Edition*.

[4] Duffy C. M., Hill A. E., Cosgrove A. P., Corry I. S., Graham H. K. (1996). Energy consumption in children with spina bifida and cerebral palsy: A Comparative Study. *Developmental Medicine and Child Neurology*.

[5] Wilmshurst S., Ward K., Adams J. E., Langton C. M., Mughal M. Z. (1996). Mobility status and bone density in cerebral palsy. *Archives of Disease in Childhood*.

[6] Lepage C., Noreau L., Bernard P.-M. (1998). Association between characteristics of locomotion and accomplishment of life habits in children with cerebral palsy. *Physical Therapy*.

[7] Damiano D. L., DeJong S. L. (2009). A systematic review of the effectiveness of treadmill training and body weight support in pediatric rehabilitation. *Journal of Neurologic Physical Therapy*.

[8] Willoughby K. L., Dodd K. J., Shields N. (2009). A systematic review of the effectiveness of treadmill training for children with cerebral palsy. *Disability and Rehabilitation*.

[9] Dodd K. J., Foley S. (2007). Partial body-weight-supported treadmill training can improve walking in children with cerebral palsy: a clinical controlled trial. *Developmental Medicine and Child Neurology*.

[10] Willoughby K. L., Dodd K. J., Shields N., Foley S. (2010). Efficacy of partial body weight-supported treadmill training compared with overground walking practice for children with cerebral palsy: a randomized controlled trial. *Archives of Physical Medicine and Rehabilitation*.

[11] Schindl M. R., Forstner C., Kern H., Hesse S. (2000). Treadmill training with partial body weight support in nonambulatory patients with cerebral palsy. *Archives of Physical Medicine and Rehabilitation*.

[12] Meyer-Heim A., Ammann-Reiffer C., Schmartz A. (2009). Improvement of walking abilities after robotic-assisted locomotion training in children with cerebral palsy. *Archives of Disease in Childhood*.

[13] Smania N., Bonetti P., Gandolfi M. (2011). Improved gait after repetitive locomotor training in children with cerebral palsy. *American Journal of Physical Medicine and Rehabilitation*.

[14] Druzbicki M., Rusek W., Snela S. (2013). Functional effects of robotic-assisted locomotor treadmill thearapy in children with cerebral palsy. *Journal of Rehabilitation Medicine*.

[15] Hidler J. M., Wall A. E. (2005). Alterations in muscle activation patterns during robotic-assisted walking. *Clinical Biomechanics*.

[16] Inman V., Ralston H. J., Todd F. (1981). *Human Walking*.

[17] Ballaz L., Robert M., Parent A., Prince F., Lemay M. (2014). Impaired visually guided weight-shifting ability in children with cerebral palsy. *Research in Developmental Disabilities*.

[18] Winter D. A., MacKinnon C. D., Ruder G. K., Wieman C. (1993). An integrated EMG/biomechanical model of upper body balance and posture during human gait. *Progress in Brain Research*.

[19] Reinkensmeyer D. J., Akoner O., Ferris D. P., Gordon K. E. Slacking by the human motor system: computational models and implications for robotic orthoses.

[20] Palisano R., Rosenbaum P., Walter S., Russell D., Wood E., Galuppi B. (1997). Development and reliability of a system to classify gross motor function in children with cerebral palsy. *Developmental Medicine and Child Neurology*.

[21] Wu M., Hornby T. G., Landry J. M., Roth H., Schmit B. D. (2011). A cable-driven locomotor training system for restoration of gait in human SCI. *Gait & Posture*.

[22] Alton F., Baldey L., Caplan S., Morrissey M. C. (1998). A kinematic comparison of overground and treadmill walking. *Clinical Biomechanics*.

[23] Zeni J. A., Richards J. G., Higginson J. S. (2008). Two simple methods for determining gait events during treadmill and overground walking using kinematic data. *Gait and Posture*.

[24] Yen S.-C., Schmit B. D., Landry J. M., Roth H., Wu M. (2012). Locomotor adaptation to resistance during treadmill training transfers to overground walking in human SCI. *Experimental Brain Research*.

[25] Liao H.-F., Jeng S.-F., Lai J.-S., Cheng C.-K., Hu M.-H. (1997). The relation between standing balance and walking function in children with spastic diplegic cerebral palsy. *Developmental Medicine and Child Neurology*.

[26] Stackhouse C., Shewokis P. A., Pierce S. R., Smith B., McCarthy J., Tucker C. (2007). Gait initiation in children with cerebral palsy. *Gait and Posture*.

[27] Dallmeijer A. J., Rameckers E. A., Houdijk H., de Groot S., Scholtes V. A., Becher J. G. (2015). Isometric muscle strength and mobility capacity in children with cerebral palsy. *Disability and Rehabilitation*.

[28] Thompson N., Stebbins J., Seniorou M., Newham D. (2011). Muscle strength and walking ability in Diplegic Cerebral Palsy: implications for assessment and management. *Gait & Posture*.

[29] Dietz V., Müller R., Colombo G. (2002). Locomotor activity in spinal man: significance of afferent input from joint and load receptors. *Brain*.

[30] Pearson K. G., Misiaszek J. E., Fouad K. (1998). Enhancement and resetting of locomotor activity by muscle afferents. *Annals of the New York Academy of Sciences*.

[31] Kao P.-C., Lewis C. L., Ferris D. P. (2010). Short-term locomotor adaptation to a robotic ankle exoskeleton does not alter soleus Hoffmann reflex amplitude. *Journal of NeuroEngineering and Rehabilitation*.

[32] Kaelin-Lane A., Sawaki L., Cohen L. G. (2005). Role of voluntary drive in encoding an elementary motor memory. *Journal of Neurophysiology*.

[33] Lotze M., Braun C., Birbaumer N., Anders S., Cohen L. G. (2003). Motor learning elicited by voluntary drive. *Brain*.

[34] van der Krogt M. M., Sloot L. H., Buizer A. I., Harlaar J. (2015). Kinetic comparison of walking on a treadmill versus over ground in children with cerebral palsy. *Journal of Biomechanics*.

[35] van der Krogt M. M., Sloot L. H., Harlaar J. (2014). Overground versus self-paced treadmill walking in a virtual environment in children with cerebral palsy. *Gait and Posture*.

[36] Reisman D. S., Wityk R., Silver K., Bastian A. J. (2009). Split-belt treadmill adaptation transfers to overground walking in persons poststroke. *Neurorehabilitation and Neural Repair*.

[37] Bar-On L., Molenaers G., Aertbeliën E., Monari D., Feys H., Desloovere K. (2014). The relation between spasticity and muscle behavior during the swing phase of gait in children with cerebral palsy. *Research in Developmental Disabilities*.

[38] Vasudevan E. V. L., Torres-Oviedo G., Morton S. M., Yang J. F., Bastian A. J. (2011). Younger is not always better: development of locomotor adaptation from childhood to adulthood. *The Journal of Neuroscience*.

